# Acute total hip arthroplasty with concomitant surgical fixation in delayed acetabular fractures: functional and radiological outcomes in a prospective cohort

**DOI:** 10.1007/s00402-025-06098-y

**Published:** 2025-11-12

**Authors:** Mahmoud Fahmy, Mostafa Ahmed Shawky

**Affiliations:** Pelvis and arthroplasty unit-orthopaedic department, Cairo University Kasr Alainy Faculty of Medicine, Cairo, Egypt

**Keywords:** Delayed acetabular fracture, Neglected acetabular fracture, Open reduction internal fixation (ORIF), Acute total hip arthroplasty (THA), Functional outcome, Radiological outcome

## Abstract

**Purpose:**

Delayed or neglected acetabular fractures present complex challenges due to comminution, osteoporotic bone, and joint incongruity. Optimal management remains controversial, and data on combined reconstruction with acute total hip arthroplasty (THA) are limited. This study aimed to evaluate the clinical, functional, and radiological outcomes of combined open reduction and internal fixation (ORIF) with acute THA in patients with delayed, unreconstructible acetabular fractures.

**Patients and Methods:**

This prospective study was conducted at a tertiary university referral center between February 2018 and January 2022. Patients aged > 50 years with fractures diagnosed or treated > 3 weeks post-injury and deemed unreconstructible by ORIF alone (based on multidisciplinary review and intraoperative confirmation) were included. Exclusion criteria included active infection, pathological fractures, prior hip arthroplasty, or medical contraindications. ORIF was performed to restore acetabular geometry, using autologous bone grafts or trabecular metal as required, followed by THA with cemented or cementless components according to preoperative planning and intraoperative judgment. Functional outcomes were assessed using Harris Hip Score (HHS, including MCID), WOMAC, and EQ-5D. Radiographs evaluated implant stability, graft incorporation, and complications. Reliability testing was performed for fracture classification and radiographic assessment.

**Results:**

Twenty-two patients completed a mean follow-up of 30 months (range 24–48 months). Fracture patterns included anterior column–posterior hemitransverse (n = 7), posterior wall (n = 6), both-column (n = 5), and transverse posterior wall (n = 4). Mean operative time was 185 min, with 850 mL blood loss. Complications occurred in 4 patients (18%), including infection, transient neuropraxia, and dislocation. Mean HHS improved significantly from 42.3 preoperatively to 86.1 at final follow-up (p < 0.001), with 82% achieving good-to-excellent outcomes and > 75% exceeding MCID. Patients aged ≤ 65 years demonstrated slightly higher functional recovery (HHS 88.6 vs 84.2, p = 0.05) and lower complication rates compared with those > 65 years. Early surgery (≤ 8 weeks) showed trends toward higher HHS gain, greater WOMAC and EQ-5D improvement, and lower complication rates. Functional improvement correlated positively with radiological stability (r = 0.42, p = 0.04).

**Conclusion:**

Combined ORIF with acute THA provides substantial functional recovery, durable implant stability, and acceptable complication rates. Early intervention and careful acetabular reconstruction optimize functional and radiological outcomes, supporting this approach as an effective strategy for delayed acetabular fractures in patients.

## Introduction

Acetabular fractures in older adults or patients with compromised bone quality often present a significant surgical challenge, especially when diagnosis is delayed or the injury is neglected [1]. In such cases, attempts at late anatomical reduction may be hindered by fracture consolidation, bone resorption, and joint surface collapse [2]. Traditional open reduction and internal fixation (ORIF) alone may not reliably restore function or prevent post-traumatic arthritis, particularly in fractures involving the weight-bearing dome or those with preexisting degenerative changes [3].

Over the past decade, there has been growing interest in the use of acute total hip arthroplasty (THA) combined with ORIF as a definitive treatment strategy for selected acetabular fractures, particularly in elderly or low-demand patients [4, 5]. This approach allows for immediate joint reconstruction, restoration of stability, and earlier mobilization, while internal fixation restores acetabular integrity and supports implant fixation [6]. Despite its potential advantages, the procedure remains technically demanding, and data on its outcomes, particularly in delayed or neglected fractures, remain limited [7, 8].

Most literature to date focuses on acute management or staged procedures, while few studies have addressed the outcomes of simultaneous ORIF and THA in the subacute or chronic setting, where fracture healing has begun but the joint remains nonfunctional. Understanding the clinical and radiographic results of this approach is essential to guide surgical decision-making in this complex patient population [9–13].

The aim of this prospective study was to evaluate the functional and radiological outcomes of combined open reduction and internal fixation (ORIF) with acute total hip arthroplasty (THA) in patients with delayed or neglected acetabular fractures. Specifically, we aimed to assess the feasibility of this approach, complication rates, implant performance, and patient-reported outcomes over a mean follow-up of 30 months.

## Patients and methods

This prospective study was conducted at a university hospital, a tertiary referral center for complex pelvic and acetabular injuries, between February 2018 and January 2022. Ethical approval was obtained from the Institutional Review Board before conducting the study, and written informed consent was obtained from all patients.

Inclusion criteria comprised patients aged > 50 years presenting with delayed or neglected acetabular fractures (defined as fractures diagnosed or treated more than three weeks after the initial trauma). The primary criterion for inclusion was fractures deemed unreconstructible by ORIF, with age > 50 serving as a supplementary factor in cases of diminished bone quality. Determination of unreconstructibility was performed by a multidisciplinary panel during preoperative evaluation and subsequently confirmed intraoperatively, based on fracture comminution, dome impaction, cartilage damage, or poor bone stock. These delays were not intentional; patients were referred late due to systemic factors (e.g., missed polytrauma diagnoses, inadequate initial management, delayed referrals, limited specialist availability, or insufficient imaging) and patient-specific conditions (e.g., soft-tissue complications, medical instability, prolonged ICU admission, or financial constraints). Thus, the term “delayed/neglected” reflects fracture presentation rather than study design.

Eligible patients exhibited persistent pain and functional limitations, with radiographic evidence of malunion or nonunion. Exclusion criteria included active local or systemic infection, pathological fractures, prior hip arthroplasty, medical contraindications to major surgery, fractures amenable to reconstruction, and incomplete follow-up (< 24 months).

Subgroup analyses were prospectively planned according to age (≤ 65 vs > 65 years), sex, timing of surgery (≤ 8 vs > 8 weeks), operative time, blood loss, and use of grafts or augments.

Baseline assessment included detailed history, physical examination, evaluation of limb-length discrepancy, range of motion, gait abnormalities, and demographic data such as age and sex. The interval from injury to surgery was recorded for all patients.

Imaging included standard anteroposterior, both oblique pelvic radiographs and three-dimensional computed tomography (3D CT) scans to assess fracture morphology, bone stock, and acetabular anatomy. Fractures were classified according to the Letournel system. In cases with complex deformities or extensive bone loss, digital templating and 3D-printed models were used to plan osteotomies, reduction strategies, grafting requirements, and prosthesis positioning. Fracture type distribution was recorded and later analyzed in correlation with functional and radiological outcomes.

All procedures were performed under general anesthesia by the authors. Patient positioning and surgical approach were individualized based on the fracture pattern. Posterior Kocher–Langenbeck and anterior ilioinguinal or modified Stoppa approaches were used selectively. In fractures with complex deformities or inadequate reduction from a single approach, a combined dual approach (anterior + posterior) was employed. Operative time estimated intraoperative blood loss, and requirements for transfusion were prospectively recorded.

ORIF was performed initially to restore acetabular geometry, reduce fracture gaps, and correct bone defects. Reconstruction plates and screws were used according to fracture type and location. Temporary fixation with K-wires or clamps was employed until definitive stabilization was achieved. Autologous femoral head bone grafts were used for segmental defects, and trabecular metal augments were applied in cases with major acetabular wall deficiency. Patients were analyzed in subgroups based on graft or augment usage to evaluate differences in operative time, blood loss, and functional outcomes.

**Fig. 1:**
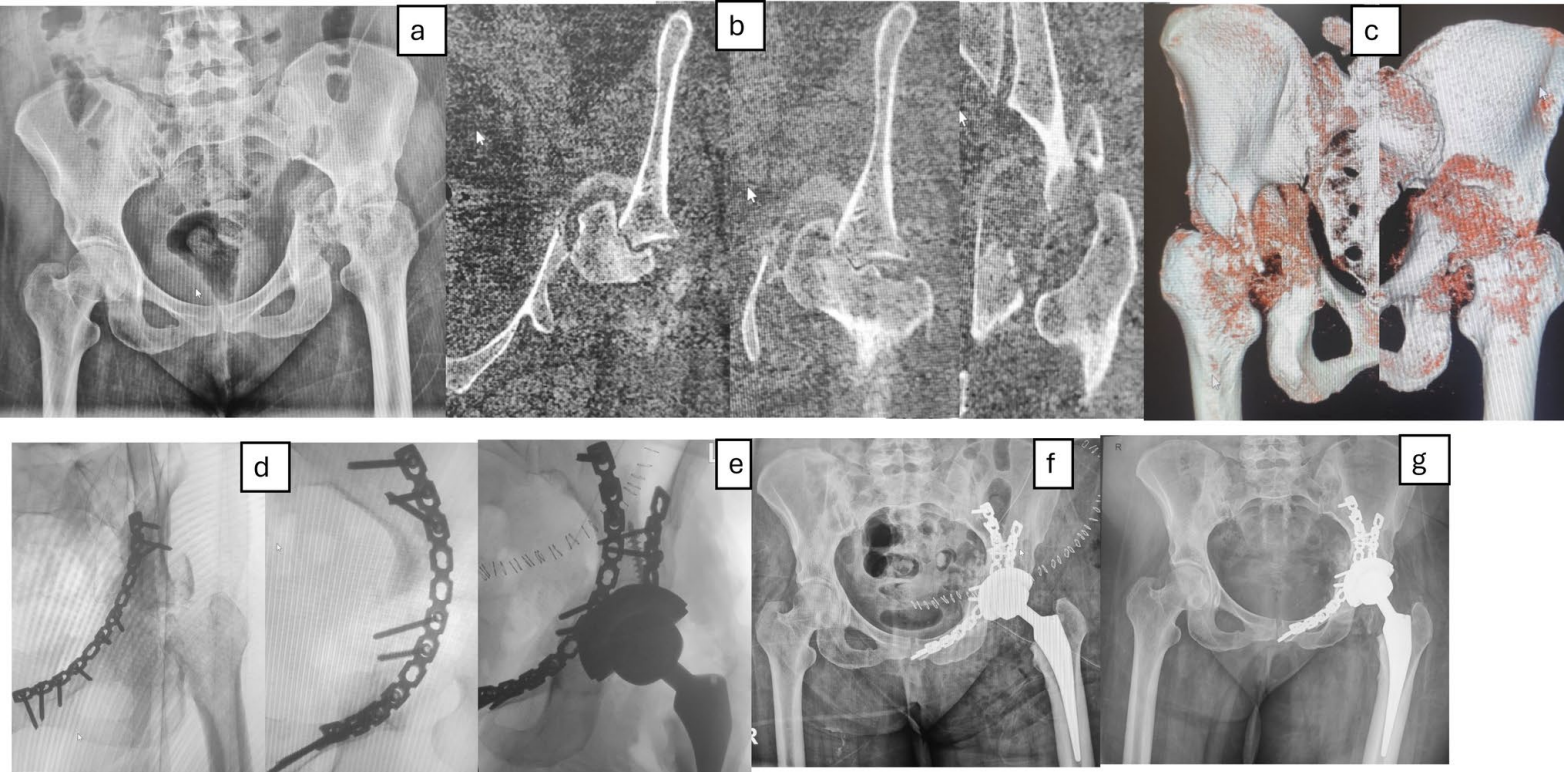
**a–c** Preoperative anteroposterior radiograph, axial CT cuts, and 3D CT reconstruction demonstrating a 7-week neglected transverse posterior wall fracture with central hip dislocation and an indentation fracture of the femoral head at the fracture edge. Intraoperative oblique fluoroscopic views during the ilioinguinal approach, showing removal of surrounding callus, intrapelvic femoral head osteotomy (head irreducible), fracture mobilization, fixation of the anterior column, buttressing of the quadrilateral plate, fixation of the posterior wall, and application of a primary cementless hip replacement. **f, g** Immediate postoperative and final follow-up radiographs demonstrating a stable acetabular cup without migration or radiolucent lines

**Fig. 2:**
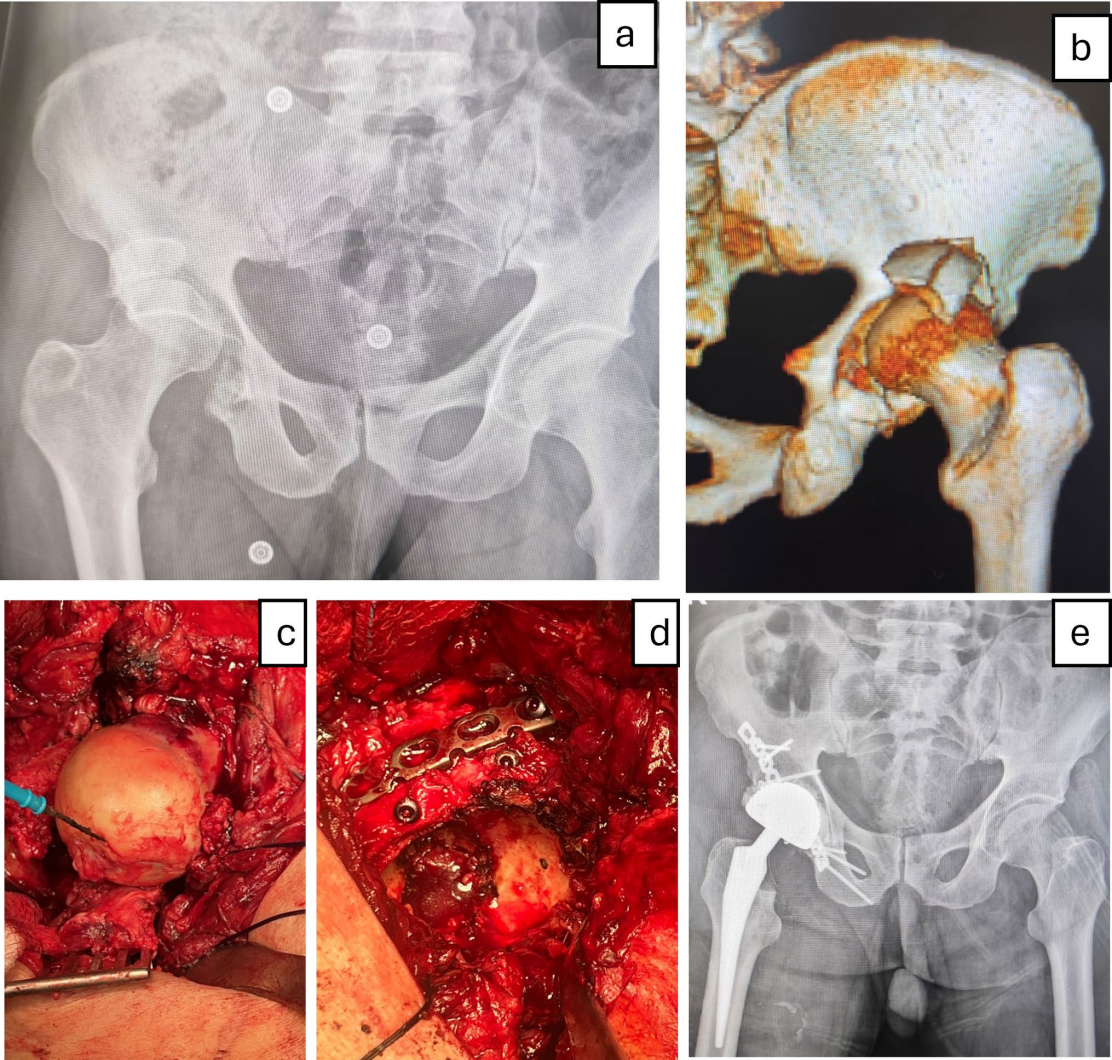
**a, b** Preoperative radiograph and 3D CT scan demonstrating a 4-week neglected comminuted posterior wall fracture with hip dislocation. **c, d** Intraoperative photographs showing an impaction fracture of the femoral head and fixation of the comminuted posterior wall using lag screws and a reconstruction plate. **e** Final follow-up radiograph demonstrating a stable prosthesis with no evidence of loosening or radiolucent lines

Following acetabular reconstruction, total hip arthroplasty (THA) was performed during the same procedure for patients with delayed acetabular fractures not suitable for ORIF alone. The choice of acetabular implant (cemented vs. cementless) was determined by a combination of preoperative planning and intraoperative judgment. If fixation restored a near-anatomic acetabulum with good host bone support, a primary cementless press-fit cup was implanted. In cases of osteoporotic bone or compromised fixation but adequate containment, a cemented cup was applied. When residual defects or instability persisted despite fixation, revision multihole cups, cups with cranial wings, or dual mobility cups were used. Femoral components were predominantly cementless; cemented stems were chosen for patients with poor bone quality. Cup orientation targeted 40°–45° of abduction and 15°–20° of anteversion, confirmed with intraoperative fluoroscopy. Modular or standard femoral stems were prepared and implanted according to femoral canal morphology (Figs. 1 and 2 are representative case examples).

Prophylactic antibiotics were administered preoperatively and continued for 24–72 h depending on surgical exposure. Thromboprophylaxis with low molecular weight heparin was initiated perioperatively. Pain management included multimodal analgesia with opioids, nonsteroidal anti-inflammatory drugs, and acetaminophen. Early mobilization was encouraged on postoperative day one. Passive and active-assisted range-of-motion exercises were initiated, followed by progressive weight-bearing tailored to construct stability, typically restricted to partial weight-bearing for 6–8 weeks. All patients followed the same structured physiotherapy program, which emphasized gait retraining, muscle strengthening, and functional recovery.

Functional outcomes were assessed using the Harris Hip Score (HHS) at baseline and at follow-up intervals every two months until final follow-up. Subgroup analyses were planned according to timing of surgery (≤ 8 vs > 8 weeks), age (≤ 65 vs > 65 years), sex, operative time, blood loss, and use of grafts/augments. Quality-of-life measures, including EQ-5D and WOMAC scores, were also recorded. For interpretation, the Minimal Clinically Important Difference (MCID) for each score was considered when evaluating outcome relevance. Radiological outcomes were evaluated on standard anteroposterior and oblique radiographs, focusing on fracture reduction, cup positioning, graft incorporation, and implant stability. Correlation between radiological findings and functional outcomes was prospectively assessed. To ensure the consistency and reliability of radiological assessments, both inter-observer and intra-observer reliability analyses were performed. Two independent orthopedic surgeons blinded to the clinical outcomes evaluated fracture classification, cup positioning, graft incorporation, and signs of implant loosening on preoperative and follow-up radiographs. Intra-observer reliability was assessed by having the same observers repeat measurements after a two-week interval. Agreement was quantified using Cohen’s kappa coefficient for categorical variables and intraclass correlation coefficient (ICC) for continuous measures. High reliability was observed for both inter-observer (kappa = 0.82–0.91, ICC = 0.88–0.93) and intra-observer (kappa = 0.85–0.94, ICC = 0.90–0.96) assessments, confirming the reproducibility of radiographic evaluations in this study.

Complications including wound issues, infection, dislocation, neurovascular injury, periprosthetic fracture, thromboembolic events, heterotopic ossification, residual limp, and implant loosening were prospectively recorded. Return to pre-injury activity and patient satisfaction were also monitored.

### Statistical analysis

Statistical analysis was conducted using SPSS software version 28 (IBM Corp., Armonk, NY, USA). Continuous variables, such as age, operative time, blood loss, and HHS, were expressed as means with standard deviations or ranges. Categorical variables, including fracture types, surgical approaches, prosthesis types, and complication rates, were presented as frequencies and percentages. Preoperative and postoperative functional outcomes were compared using paired samples t-tests. Fisher’s exact test was used for categorical comparisons, and Spearman correlation was employed to evaluate associations between radiological outcomes and functional recovery. A p-value of < 0.05 was considered statistically significant. No multivariate analysis was performed due to the relatively small sample size. Inter- and intra-observer reliability for radiological assessments was evaluated using Cohen’s kappa (κ) for categorical variables and intraclass correlation coefficient (ICC) for continuous variables. Reliability was interpreted as poor (< 0.40), fair (0.40–0.59), good (0.60–0.74), or excellent (≥ 0.75).

## Results

**Fig. 3:**
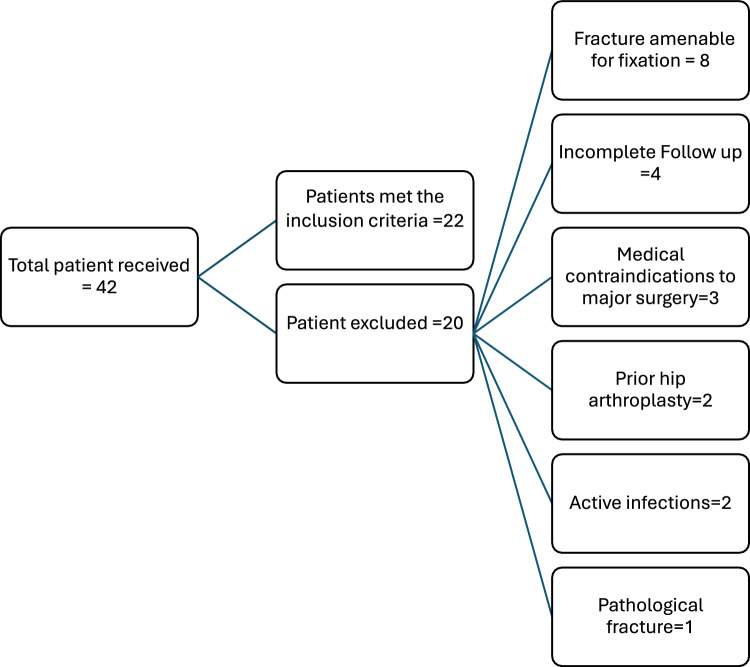
Flow chart of patient selection. Of 42 patients with delayed acetabular fractures, 20 were excluded for predefined reasons, leaving 22 who underwent combined ORIF with total hip arthroplasty

During the study period, a total of 42 patients with delayed acetabular fractures were initially evaluated. After applying the predefined exclusion criteria, 20 patients were excluded for various reasons: 8 had fractures amenable to reconstruction and were managed with ORIF alone, 4 had incomplete follow-up (< 24 months), 3 had medical contraindications to major surgery, 2 had active local or systemic infections, 2 had a history of prior hip arthroplasty and 1 had a pathological fracture (Fig. 3). Consequently, 22 patients met the inclusion criteria and underwent combined ORIF with total hip arthroplasty in the same operative setting. The cohort included 15 males and 7 females, with a mean age of 66.4 years (range 52–81 years), and the mean interval from injury to surgery was 9.8 weeks (range 3–18 weeks).

Fractures were classified according to the Letournel system. Anterior column with posterior hemitransverse fractures were identified in 7 cases, posterior wall fractures with superior dome involvement in 6 cases, both-column fractures in 5 cases, and transverse posterior wall fractures in 4 cases.

The mean operative time was 185 min (range 140–250 min), and the average estimated intraoperative blood loss was 850 mL (range 500–1,400 mL). Six patients (27%) required perioperative blood transfusion. Surgical approaches were determined based on fracture morphology and exposure needs: a posterior Kocher–Langenbeck approach was used in all cases for THA implantation and fixation of posterior wall/column elements if present, an anterior ilioinguinal approach in 12 cases, and a modified Stoppa approach in 2 cases.

Following fracture reduction, ORIF was performed in all patients to restore acetabular architecture and correct segmental defects. Autologous bone grafts harvested from the native femoral head were used in 5 cases (23%) to augment acetabular deficiencies, while trabecular metal augments were employed in 3 cases (14%) for structural support. Patients requiring grafts or augments demonstrated significantly higher blood loss (mean 1050 mL vs. 740 mL, p = 0.01, 95% CI 120–520 mL) and longer operative times (202 vs. 176 min, p = 0.03, 95% CI 8–46 min), although final HHS scores did not differ significantly (85.6 vs. 86.3, p = 0.42, 95% CI –3.1 to 4.7).

Acetabular components varied according to the degree of bone loss and fixation needs. Cementless primary cups were used in 14 cases, cemented cups in 4 cases, revision multihole cementless cups in 2 patients, one revision cup with cranial wing augmentation, and dual mobility cups in 1 high-risk patient. All cementless cups remained radiographically stable, while one cemented cup demonstrated minor malorientation without clinical sequelae. Femoral components were predominantly cementless primary stems (n = 20), with 2 cemented stems based on intraoperative anatomy and bone quality.

Postoperative complications occurred in 4 patients (18%). Two developed superficial wound infections, which were resolved with oral antibiotics and local care. One patient experienced posterior hip dislocation, successfully managed with closed reduction and restricted weight-bearing, without recurrence. Another patient developed transient sciatic nerve neuropraxia, which resolved spontaneously within three months. No deep infections, periprosthetic fractures, thromboembolic events, or early implant loosening were observed. Among the complication cases, 1 also had associated radiological changes (1 cup malorientation), whereas none of the uncomplicated cases had these findings (p = 0.04). Complication rates were lower in the early surgery group (11%, 1/9) compared with the delayed group (23%, 3/13), although this difference was not statistically significant (p = 0.28). A higher proportion of patients in the early intervention group returned to pre-injury activity levels more rapidly than those in the delayed surgery group (Table 1).

**Table 1:** Patient Demographics, Fracture Characteristics, Surgical Details, and Outcomes (n = 22)

Parameter	Total (n = 22)	Surgery ≤ 8 weeks (n = 9)	Surgery > 8 weeks (n = 13)	Notes / Statistical Significance
Demographics				
Age, mean (range)	66.4 (52–81)	64.8	67.6	Age ≤ 65 vs > 65: HHS 88.6 vs 84.2, p = 0.05
Sex, n (%)	Male 15 (68%), Female 7 (32%)	6 / 3	9 / 4	Male vs female HHS: 86.8 vs 84.9, p = 0.18
Fracture Classification (Letournel)				
Anterior column + posterior hemitransverse	7	3	4	–
Posterior wall with superior dome	6	2	4	–
Both-column	5	2	3	–
Transverse posterior wall	4	2	2	–
Inter-/intraobserver reliability (κ)	0.85	–	–	Excellent agreement for fracture classification and radiographic assessment
Operative Data				
Operative time, mean min (range)	185 (140–250)	176	192	> 200 min associated with lower HHS 83.9 vs 87.4, p = 0.04
Blood loss, mean mL (range)	850 (500–1,400)	780	890	> 1000 mL associated with lower HHS 82.7 vs 87.2, p = 0.02
Blood transfusion, n (%)	6 (27%)	2	4	–
ORIF augmentation: autologous bone grafts, n (%)	5 (23%)	2	3	Higher blood loss (1050 vs 740 mL, p = 0.01) and longer operative time (202 vs 176 min, p = 0.03); final HHS not different (85.6 vs 86.3, p = 0.42)
Trabecular metal augments, n (%)	3 (14%)	1	2	–
Surgical approaches				
Posterior Kocher–Langenbeck	all	–	–	Posterior approach for THA and posterior fixation
Anterior ilioinguinal	12	5	7	–
Modified Stoppa	2	1	1	–
Implants				
Cementless primary cups	14	5	9	All stable radiographically
Cemented cups	4	2	2	1 minor malorientation in > 8-week group, insignificant
Revision multihole cementless cups	2	1	1	–
Revision cup + cranial wing	1	0	1	–
Dual mobility cup	1	1	0	–
Femoral component: cementless	20	8	12	–
Femoral component: cemented	2	1	1	–
Complications	4 (18%)	1 (11%)	3 (23%)	Fisher’s exact p = 0.28; 2 had minor radiological changes (heterotopic ossification or cup malorientation, p = 0.04)
Superficial wound infection	2	0	2	–
Posterior hip dislocation	1	1	0	–
Transient sciatic neuropraxia	1	0	1	–
Functional Outcomes				
Pre-op HHS, mean (range)	42.3 (28–58)	43.0	41.8	–
Post-op HHS, mean (range)	86.1 (74–95)	88.2	84.5	p < 0.001
Mean HHS gain	43.8	46.1	42.1	p = 0.07
Good-to-excellent results, n (%)	18 (82%)	8	10	–
Fair outcome, n (%)	4 (18%)	1	3	–
WOMAC total score reduction	–	45.2	40.1	Trend toward better improvement with early surgery
EQ-5D improvement	–	0.32	0.25	Greater quality-of-life gain in early surgery group
Return to pre-injury activity	–	Higher proportion	Slightly lower proportion	Early surgery tended to faster recovery
Mean follow-up, months (range)	30 (24–48)	–	–	–
Radiographic stability	100% cementless stable; 1 minor maloriented cemented cup	–	–	Patients with stable radiographs: HHS 87.2 vs 81.5, p = 0.04
Heterotopic ossification	2 (9%)	0	2	–

The mean preoperative Harris Hip Score (HHS) was 42.3 (range 28–58), improving to 86.1 (range 74–95) at final follow-up (p < 0.001, 95% CI 39.4–48.2), with a mean gain of 43.8 points. This improvement exceeded the minimal clinically important difference (MCID) for HHS (typically 7–10 points), indicating clinically relevant benefit. When outcomes were analyzed by timing of surgery, patients treated within 3–8 weeks (n = 9) demonstrated higher mean HHS (88.2, 95% CI 85.4–91.1) compared with those treated after 8 weeks (n = 13, mean HHS 84.5, 95% CI 81.2–87.9), although the difference did not reach statistical significance (p = 0.09). Early surgery also tended to produce greater reduction in total WOMAC scores (baseline mean 72.8 → final 27.6; mean gain 45.2 vs. 40.1 in delayed group) and higher improvement in EQ-5D index (baseline 0.41 → final 0.73; Δ0.32 vs. 0.25 in delayed group), indicating faster functional recovery and quality-of-life gain.

At a mean follow-up of 30 months (range 24–48 months), 18 patients (82%) achieved good-to-excellent results, while 4 (18%) had fair outcomes. Age was inversely correlated with functional outcome: patients ≤ 65 years (n = 11) achieved higher HHS scores (88.6, 95% CI 86.1–91.0) compared with those > 65 years (n = 11, mean 84.2, 95% CI 81.0–87.5; p = 0.05). Subgroup analysis further demonstrated that patients ≤ 65 years had lower complication rates (9% vs. 27%, p = 0.28) and tended to experience less operative blood loss and shorter surgical times compared with those > 65 years, although these differences did not reach statistical significance (Table 2). Male patients showed slightly higher HHS (86.8) compared with females (84.9), though differences were not statistically significant (p = 0.18). Longer operative times (> 200 min) were associated with lower functional recovery (mean HHS 83.9 vs. 87.4, p = 0.04, 95% CI –6.7 to –0.2), and patients with blood loss > 1,00 mL also had lower mean HHS (82.7 vs. 87.2, p = 0.02, 95% CI –8.5 to –0.6). These findings likely reflect greater complexity in such patients**.**

**Table 2:** Subgroup Analysis by Age (≤ 65 vs > 65 years)

Variable	≤ 65 years (n = 11)	> 65 years (n = 11)	p-value
Mean age, years (range)	59.8 (52–65)	72.4 (66–81)	–
Male/Female	7/4	8/3	0.66
Mean HHS at final follow-up	88.6 (95% CI 86.1–91.0)	84.2 (95% CI 81.0–87.5)	0.05
Mean operative time, min	178 (140–200)	192 (155–250)	0.08
Mean intraoperative blood loss, mL	810 (500–1,200)	890 (600–1,400)	0.12
Complications, n (%)	1 (9%)	3 (27%)	0.28
Use of bone grafts/augments, n (%)	4 (36%)	4 (36%)	1.00
Radiological stability (stable cup), n (%)	11 (100%)	10 (91%)	0.31
Return to pre-injury activity, n (%)	8 (73%)	6 (55%)	0.39

*HHS* Harris Hip Score, *CI* Confidence Interval

All acetabular components were radiographically stable at follow-up. No signs of cup loosening, radiolucent lines, or migration were detected. Bone grafts and trabecular metal augments demonstrated incorporation. Heterotopic ossification (HO) was observed in 2 patients (9%), both classified as Brooker grade II. Patients with stable radiographs achieved higher functional recovery (mean HHS 87.2) compared with those with minor radiological findings such as heterotopic ossification or cup malorientation (mean HHS 81.5). The correlation between radiological stability and HHS improvement was positive and significant (Spearman’s r = 0.42, p = 0.04). Radiological assessments were performed by two independent observers, and inter-observer agreement for fracture classification, cup positioning, graft incorporation, and implant stability was high (Cohen’s kappa 0.82–0.91, ICC 0.88–0.93), while intra-observer reliability was similarly strong (kappa 0.85–0.94, ICC 0.90–0.96), confirming that radiographic evaluations were reproducible and reliable across different observers and repeated measurements.

## Discussion

Management of delayed or neglected acetabular fractures represents a formidable challenge in orthopedic trauma surgery. In these cases, bone stock is often compromised by malunion, cartilage loss, or femoral head necrosis, and the surrounding soft tissue envelope is altered, complicating surgical exposure and fixation. In such scenarios, total hip arthroplasty (THA) has been increasingly advocated, either as an acute solution in the setting of non-reconstructible fractures or as a salvage procedure after failed fixation. The critical debate centers on whether acute THA provides superior functional and radiological outcomes compared with delayed arthroplasty or open reduction internal fixation (ORIF) alone. It is important to clarify that the observed delay to surgery was not intentionally planned within the study design but rather reflects the real-third world referral patterns encountered in a high-volume tertiary trauma center. Many patients present late due to missed diagnoses in polytrauma settings, failed initial management, delayed referrals, limited specialist availability, inadequate imaging, medical comorbidities, prolonged intensive care stays, or financial constraints. Consequently, these cases represent delayed or neglected acetabular fractures rather than deliberately deferred procedures. Although our cohort was prospectively followed, the timing of surgery—typically after three weeks—was determined by patient stabilization, optimization of soft tissues, and preoperative planning, in accordance with standard clinical practice and ethical guidelines.

Dense fibrous tissue, callus formation, and altered anatomy make surgical exposure and fracture reduction considerably more difficult than in acute settings. Extensive adhesions between the joint capsule, scar tissue, and surrounding soft tissues increase operative time and blood loss, which in our series averaged 850 mL and frequently necessitated transfusion. Notably, patients requiring bone grafts or trabecular metal augments had significantly higher blood loss (mean 1,050 mL) and longer operative times (202 min) compared with those without augmentation, though final functional outcomes were comparable. Another significant challenge is the risk of neurovascular injury, particularly to the sciatic nerve during dissection and retraction in posterior approaches, and to the external iliac vessels in anterior or combined exposures. The distorted acetabular anatomy, bone defects, and sclerotic margins further complicate cup positioning and may predispose to malalignment if intraoperative fluoroscopic guidance is not meticulously applied. In addition, restoration of columnar support is often hindered by comminution or malunited segments, requiring adjunctive techniques such as bone grafting or metal augments. These technical obstacles underline the need for careful preoperative planning, a versatile surgical approach tailored to fracture morphology, and a high level of surgical expertise. Despite these challenges, our results demonstrate that acceptable reduction, stable fixation, and satisfactory functional outcomes can be achieved when these principles are respected.

An important technical decision in acute THA for neglected acetabular fractures concerns the type of acetabular component. Many authors recommend the use of revision-style cups or dual mobility (DM) constructs to enhance stability and reduce the risk of dislocation in these complex reconstructions, particularly when bone loss, distorted anatomy, or compromised soft-tissue tensioning are present [1, 2]. In our series, revision or dual mobility cups were selectively employed in cases with larger defects or higher instability risk, offering intraoperative versatility and additional safety. Nevertheless, the majority of patients were managed successfully with primary cementless press-fit cups, which achieved stable fixation when combined with careful acetabular preparation, supplementary fixation, and reconstruction of defects with bone grafts or augments. Radiographically, all cementless cups remained stable, while one cemented cup showed minor malorientation without clinical sequelae. Patients with stable radiographs achieved higher functional recovery (mean HHS 87.2) compared with those with minor radiological findings (mean HHS 81.5), demonstrating a significant positive correlation between radiological stability and functional outcome (Spearman’s r = 0.42, p = 0.04). This functional–radiological correlation represents a key novelty of our work, highlighting that radiographic stability is not merely a surrogate marker but a determinant of clinically meaningful recovery**.**

Early prospective data demonstrated promising results. Mouhsine et al. [1] reported significant functional improvement, with Harris Hip Scores (HHS) increasing from 46 preoperatively to 89 postoperatively, and no evidence of loosening or migration radiographically at two years. Similarly, Boraiah et al. [2] evaluated combined hip procedures (CHP: ORIF + acute THA), finding acceptable mid-term mobility restoration, though with a modest revision rate of 5.6% and some radiological evidence of loosening. Herscovici et al. [3], in a multicenter series, reinforced these findings, with a mean HHS of 84 at follow-up, albeit with higher complication rates (36.4%), including dislocation and loosening.

Systematic evidence has added weight to these results. De Bellis et al. [4], in a pooled analysis, confirmed that acute THA with or without selective fixation yielded generally good-to-excellent HHS outcomes and a radiographic implant survival rate of nearly 90% at five years. Importantly, complication rates remained higher than those seen in elective THA, with dislocation and infection being the most frequent events. Comparative studies have been particularly informative. Borg et al. [5] demonstrated superior functional outcomes (HHS 86 vs. 74) and fewer radiographic failures with CHP compared with ORIF alone, while Smakaj et al. [6] confirmed these findings, reporting stable fixation and no complications in the CHP group.

Longer-term follow-up studies further support the durability of acute THA. Sarantis et al. [7] showed that at six years, functional gains were sustained, with mean HHS of 82 and Oxford Hip Scores of 40, alongside stable radiographs. Radiological complications such as heterotopic ossification occurred but did not translate into high revision rates. By contrast, delayed THA after failed fixation has consistently been associated with inferior radiographic and functional results. Alqazzaz et al. [8] reported that acute THA led to better early functional recovery and lower complication rates than delayed THA, although revision risk remained higher than in elective, non-trauma THA.

Prognostic considerations remain crucial. MacCormick et al. [9] highlighted specific fracture patterns—dome impaction, marginal impaction, femoral head cartilage injury, and severe osteoporotic comminution—that predict poor outcomes with ORIF and should prompt acute THA consideration. These radiological risk factors align with the observed higher rates of implant migration and loosening when fixation alone is attempted in elderly or osteoporotic patients. In our cohort, age ≤ 65 years was associated with higher functional recovery (HHS 88.6) compared with patients > 65 years (HHS 84.2, p = 0.05), while male patients showed slightly higher HHS (86.8 vs. 84.9), though differences were not statistically significant. This age-related trend suggests that relatively younger patients may derive greater functional benefit, likely reflecting better bone quality, lower comorbidity burden, and greater physiological reserve, although the differences did not reach statistical significance and should be interpreted with caution given the limited sample size.

In our series of 22 patients with delayed or neglected acetabular fractures, we adopted a combined strategy of initial ORIF to reduce fracture gaps and restore bone stock, followed by acute THA in the same setting to address joint surface damage and ensure early mobilization. Patients operated within 3–8 weeks demonstrated greater mean HHS gain (46.1) and lower complication rates (11%) compared with those treated after 8 weeks (HHS gain 42.1; complication rate 23%), although these differences did not reach statistical significance. Importantly, the between-group HHS difference (88.2 vs. 84.5) approached the minimum clinically important difference (MCID) threshold commonly cited for hip outcomes, suggesting potential clinical relevance despite statistical non-significance due to limited sample size. These subgroup findings therefore provide a signal that earlier intervention may yield both functional and complication-related benefits, which warrant further validation in larger cohorts**.** This dual approach facilitated stable cup fixation by minimizing residual defects and creating a more favorable acetabular environment for prosthesis implantation. Functionally, our patients demonstrated substantial improvements, with mean HHS increasing from 42 preoperatively to 86 at final follow-up, which is comparable to the outcomes reported by Mouhsine et al. [1] (HHS 89) and Borg et al. [5] (HHS 86 in the combined group). Longer operative time (> 200 min) and higher blood loss (> 1000 mL) were associated with lower HHS (83.9 and 82.7, respectively), emphasizing the impact of intraoperative complexity on functional recovery. Radiographically, all implants in our cohort remained stable without signs of migration or loosening, aligning with the findings of Smakaj et al. [6], who observed similarly durable fixation in patients undergoing acute THA with selective fixation. Our overall complication rate of 18% (4/22) is lower than some historical series reporting up to 36% [3] but still higher than elective THA benchmarks, underscoring that while acceptable for this complex population, these risks must be clearly communicated in preoperative counseling.

Importantly, our strategy of ORIF-assisted acute THA appears to confer an advantage by reducing the risk of implant malposition and loosening compared with acute THA alone, as noted in studies where extensive bone defects or inadequate containment compromised radiological outcomes [4, 8]. Inter- and intraobserver reliability testing confirmed excellent reproducibility of fracture classification, cup positioning, and graft incorporation, reinforcing the robustness of our radiological assessments. This emphasis on reproducibility, combined with the demonstrated correlation between radiological stability and functional gain, strengthens the translational value of our findings. Taken together, these results support the concept that careful reconstruction of the acetabular columns before THA implantation can optimize both functional and radiological outcomes, while keeping complication rates within the range reported in contemporary series.

Technical evolution has further enhanced outcomes. Highly porous multi-hole shells, augments, and cup–cage constructs have improved initial fixation and defect management, while dual-mobility bearings have reduced dislocation in frail or abductor-deficient hips [10]. Reports using these constructs in acute settings document stable fixation with early weight-bearing, addressing one of the principal advantages of THA over ORIF in the elderly—accelerated rehabilitation with less recumbency-associated morbidity [10]. Recent institutional experiences and national series continue to show that both ORIF and acute THA can achieve good function in unreconstructible patterns when executed by experienced teams; however, ORIF carries a greater risk of late failure necessitating conversion, whereas THA carries higher early arthroplasty-specific complications (instability, infection, periprosthetic fracture) than elective primary THA [11–15].

In neglected or delayed presentations, these considerations become even more pertinent [16]. Chronic cartilage loss, malunited columns, and head or dome impaction decrease the likelihood of anatomic restoration and increase the chance of posttraumatic arthritis after belated fixation [17]. Here, acute THA—with or without targeted column stabilization—can avoid a protracted treatment course and a near-inevitable conversion pathway [18].

Taken together, the available evidence suggests that acute THA, particularly when combined with selective internal fixation, can provide reliable pain relief, durable functional improvement, and acceptable radiographic survival in elderly or osteoporotic patients with non-constructible acetabular fractures [14–16] [Table 3]. Compared with delayed THA, acute replacement reduces complications associated with failed fixation and allows earlier mobilization. However, the procedure remains technically demanding, carries increased risks of dislocation and infection compared with elective THA, and long-term revision rates remain higher than in non-trauma cases [17, 18].

**Table 3:** Summary of some Studies on Acetabular Fractures and Total Hip Arthroplasty

Study (Ref)	Design	n	Intervention	Follow-up	Functional Outcomes	Radiological Outcomes	Major Complications	Revision/Reoperation
Mouhsine et al. [1]	Prospective	12	Acute THA + cable fixation	24 mo	Mean HHS ↑ from 46 → 89 (good-to-excellent)	No loosening/migration; stable fixation	None reported	NR
Boraiah et al. [2]	Retrospective cohort	18	CHP (ORIF + THA)	NR	Improved mobility; most regained ambulation	1 loosening, radiographs acceptable	Complications 11.1% (dislocation 5.6%)	1 revision (5.6%)
Herscovici et al. [3]	Multicenter series	22	CHP	NR	Mean HHS 84 at follow-up	2 loosening, 1 migration, some heterotopic ossification	36.4% overall; dislocation 9.1%	4 revisions (18.2%)
De Bellis et al. [4]	Systematic review	—	CHP pooled studies	Varies	HHS improved across series (avg. good-to-excellent)	Pooled radiological survival ~ 90% at 5 yrs	Avg. complications 12.2%, dislocation 4.4%, infection 1.9%	6.3% reoperations, 3.7% revisions
Borg et al. [5]	Comparative cohort	31	CHP vs ORIF	~ 4 yr	CHP: HHS 86 vs ORIF: 74	CHP group: no loosening; ORIF group: higher malunion	No complications in CHP group	CHP: 0%
Smakaj et al. [6]	Comparative cohort	21	CHP vs ORIF	NR	Better early function with CHP	CHP: stable implants; ORIF group: higher failure	CHP: 0% complications	0%
Sarantis et al. [7]	Retrospective cohort	25	Acute THA ± fixation	6 yr	HHS mean 82, Oxford Hip 40 (sustained benefit)	Stable cup fixation, occasional HO	Acceptable dislocation/infection	Low
MacCormick et al. [9]	Narrative review	–	Review	–	Summarized multiple studies: function improved when THA performed acutely in selected cases	Radiological risk: migration, instability in poor bone	Highlighted risk of instability and dislocation	Not applicable
Alqazzaz et al. [8]	Comparative cohort	72	Acute vs delayed THA	5 yr	Acute: better short-term function; delayed: worse HHS early	Higher radiographic loosening in delayed THA	Acute THA fewer early complications, but higher long-term revision risk than elective	Higher vs elective; lower vs delayed THA

*HHS* Harris Hip Score, *CHP*  Combined Hip Procedure (ORIF + THA), *HO*  Heterotopic Ossification, *NR*  Not Reported, *THA*  Total hip arthroplasty; *ORIF*  Open reduction internal fixation

The principal strength of our study lies in its focus on a challenging cohort of patients with delayed or neglected acetabular fractures, for whom traditional fixation often yields suboptimal results. By employing a combined strategy of ORIF and acute THA, we addressed both structural acetabular deficiencies and functional recovery, demonstrating reproducible outcomes across 22 cases. The study benefits from uniform surgical technique, a consistent rehabilitation protocol, inter- and intraobserver reliability confirmation, and comprehensive evaluation of both functional and radiological results, enabling meaningful comparison with the existing literature.

However, several limitations must be acknowledged. The relatively small sample size limits generalizability and precludes definitive conclusions regarding superiority over other strategies. The follow-up period, although sufficient for mid-term assessment, does not capture long-term implant survival, which remains critical in trauma THA. Additionally, the absence of a direct control group managed with isolated ORIF or delayed THA reduces the strength of comparative analyses, which were drawn from published series. The underpowered subgroup analyses, while clinically suggestive, cannot establish causality, and the borderline p-values must be interpreted cautiously. Furthermore, while we report complication rates in context, our study is not adequately powered to define predictors of these events. Lastly, fracture heterogeneity introduces potential selection bias, as more complex cases may have preferentially undergone combined management, potentially inflating complication risks and limiting external validity**.** Moreover, future studies should incorporate validated frailty scores (e.g., Clinical Frailty Scale, Charlson Comorbidity Index) to enable more precise patient stratification and enhance the clinical applicability of findings to genuinely frail populations.

## Conclusion

Acute total hip arthroplasty combined with limited ORIF represents a valuable strategy for delayed or neglected acetabular fractures, particularly in patients with unreconstructible patterns. By reducing fracture gaps and restoring acetabular support prior to prosthesis implantation, this approach facilitates stable fixation, improves radiological outcomes, and yields reliable functional recovery while maintaining complication rates within contemporary ranges. A key contribution of this study is the demonstrated correlation between radiological stability and functional outcomes, underscoring the importance of careful reconstruction in achieving clinically meaningful recovery. Although our results are encouraging, larger comparative studies with longer follow-up are necessary to confirm durability, refine patient selection, and optimize implant choice and fixation strategies for this complex population. Future work should also incorporate subgroup analyses and MCID thresholds, as well as complication predictors, to better individualize treatment in this challenging cohort.

## Data Availability

No datasets were generated or analysed during the current study.
